# Unlocking Deeper Insights into Medical Images with Machine Learning

**DOI:** 10.3390/bioengineering12050451

**Published:** 2025-04-25

**Authors:** Xiangxue Wang, Cheng Lu, Jun Xu

**Affiliations:** 1 Jiangsu Key Laboratory of Intelligent Medical Image Computing, Nanjing University of Information Science and Technology, Nanjing 210044, China; jxu@nuist.edu.cn; 2 Guangdong Provincial People’s Hospital (Guangdong Academy of Medical Sciences), Southern Medical University, Guangzhou 510080, China; lucheng@gdph.org.cn

Recent years have witnessed remarkable progress at the intersection of medical imaging, biochemical assays, image analysis, and machine learning. These advancements, as highlighted in our call for this Special Issue, unlock unprecedented opportunities to investigate complex and previously intractable diseases, such as cancer. Artificial intelligence (AI) is increasingly applied to multi-modal medical images, significantly boosting the objectivity and efficiency of tasks like tumor delineation and cell quantification. However, the ultimate aim extends beyond automating repetitive tasks; it lies in providing clinicians with reproducible, quantitative second opinions, predicting patient prognosis at early stages, and suggesting potential therapy responses to enable truly personalized medicine.

Achieving this goal necessitates the development of trustworthy AI tools capable of analyzing diverse medical image modalities for disease prognosis and treatment response prediction. Complex diseases often require integrating multi-scale and multi-modal images—from microscopy and histopathology to radiology and functional imaging—to capture the full landscape from the micro to the macro scale. This Special Issue, “Machine-learning-driven medical image analysis”, brings together a collection of high-quality research articles and comprehensive reviews that showcase the power and potential of this rapidly evolving field.

Several articles underscore the power of advanced imaging modalities and analytical techniques. Yang et al. [1] demonstrate the utility of high-resolution multi-functional optical coherence tomography in adult zebrafish, a valuable model system. Their work provides rich intra- and extracranial imaging outcomes, including pigmentation distribution, tissue-specific information, cranial vascular imaging, and monitoring of traumatic brain injury. Notably, they are the first to observe novel channels through cranial sutures, potentially linking intra- and extracranial vasculatures, showcasing how advanced imaging can yield fundamental biological insights.

Another significant theme from this collection is the strength of multimodal data integration and fusion, particularly for complex neurological conditions. Fu et al. [2] propose a multimodal classification framework using hypergraph latent relation learning to diagnose end-stage renal disease associated with mild cognitive impairment. By effectively combining fMRI-derived brain functional networks with cerebral blood flow data from ASL, their approach captures high-order relationships, achieves high classification accuracy, and identifies key discriminative brain regions. Similarly, Coluzzi et al. [3] compare a novel graph convolutional network using structural connectivity (based on diffusion MRI, dMRI) against the established ResNet18 using structural MRI (sMRI) for Alzheimer’s Disease (AD) classification. Their work emphasizes explainable AI (XAI), demonstrating that both models align with known AD biomarkers (MTL atrophy for sMRI, DMN alterations for dMRI) and suggesting that these different imaging perspectives offer complementary information for diagnosis.

The translation of AI into specific clinical applications is another key focus. Hernandez Torres et al. [4] evaluate various deep learning architectures for interpreting point-of-care ultrasound images in the context of eFAST exams for trauma triage. Their evaluation of models like MobileNetV2 and DarkNet53 for detecting pneumothorax, hemothorax, and abdominal hemorrhage highlights the potential for automating critical diagnostic tasks in emergency settings, although challenges remain for certain views, like bladder hemorrhage detection. Hu et al. [5] present FundusNet, an ensemble deep-learning model using retinal fundus images for the rapid and cost-effective diagnosis of neurodegenerative diseases like Parkinson’s and multiple sclerosis, alongside eye diseases. Their use of Grad-CAM for model interpretation helps identify clinically relevant retinal structures, bridging the gap between AI prediction and clinical understanding.

Addressing challenges in model training and robustness is crucial for clinical adoption. Ding and Li [6] tackle the prevalent issue of data scarcity in medical image segmentation through a semi-supervised approach incorporating curriculum consistency learning and multi-scale contrastive constraint. Their dynamic patch-based strategy, inspired by human curriculum learning, coupled with contrastive loss, significantly improves segmentation performance on polyp and skin lesion datasets. Zhou et al. [7] address the problem of inter-individual variability and outliers in sleep stage classification using EEG data. Their proposed E-SCNN model employs an ensemble strategy, combining a clustering module to group individuals based on feature similarity with a sequential CNN, thereby enhancing model robustness at the individual level.

Finally, the Special Issue is enriched by comprehensive reviews and forward-looking perspectives that contextualize the research and point towards future directions. Lee [8] provides an exhaustive review of recent deep learning advancements (2019–2023) using whole slide images for cancer prognosis, covering a wide array of methodologies and cancer types. Singh et al. [9] offer a systematic review of emerging trends (2018–2023) in fast MRI reconstruction using deep learning on undersampled k-space data, detailing progress in network architectures, training strategies, and various applications. Looking towards future paradigms, Sun et al. [10] present a perspective on the Digital Twin (DT) concept as a potential solution for personalized diagnosis and treatment in musculoskeletal diseases. They argue that DTs, integrating AI and real-time data, can overcome the limitations of traditional biomechanical analyses, enabling dynamic, individualized assessments.

The articles assembled in this Special Issue demonstrate the dynamism and potential of machine learning-driven medical image analysis. From novel imaging applications and multimodal fusion techniques to innovative training strategies and explainability methods, the research presented here pushes the boundaries towards more accurate, robust, and clinically translatable AI tools. These contributions collectively advance the goal of leveraging AI not just for automation, but for generating deeper insights into disease mechanisms and providing powerful decision support for personalized medicine across diverse fields, including neurology, oncology, trauma care, ophthalmology, and orthopedics. We extend our sincere gratitude to all authors for their invaluable contributions, and hope this collection inspires continued innovation in this exciting field.
